# Good Men in Bad Company—an Explanation Which Explains

**Published:** 1905-08

**Authors:** G. Frank Lydston

**Affiliations:** Chicago


### Abstracts and Selections.

---

### "Good Men in Bad Company—an Explanation Which Explains."

---

Chicago, December 15, 1903.

*To the Editor of the Journal of the A. M. A.:*

Dear Sir: Under the above caption, in the *Journal* of December 12th, appears a letter from Dr. John A. Wyeth, repudiating the announcement by the *Medical Brief* that he had contributed an article to its columns on fracture of the radius. He explains that the article was one which he gave to a starving female stenographer, who said she could sell it for $20, and who agreed to submit it to the *Medical Record* or *New York Medical Journal.* Failing the acceptance of the article by one or the other of these publications, it was to be returned to Dr. Wyeth. Dr. Wyeth further stated that the same woman had imposed upon other members of the New York profession in like manner. You comment upon Dr. Wyeth's letter, as follows, viz.: "Does this explain why other reputable men appear as contributors to the above so-called medical journal, in some instances with photographs?"

Will you, oh saintly editor of the purest publication known to medical literature, permit me to say a few words in reply? And will you refrain from using the blue pencil, even at the risk of being considered pride-swollen by the foregoing adjectives?

In the first place, why should Dr. Wyeth feel called upon to apologize for or explain the publication of his article in the *Medical Brief?* There are but three motives which justify the publication of most medical articles, viz., Self-improvement, self-advertisement, and the education of the medical "rank and file." We will leave out of consideration the discoverer of a new idea that shall save humanity from suffering and death. He is such a rare bird that he scarcely counts in the present issue. Pray inform me, Mr. Editor, which of the desiderata mentioned are not conserved by the writing of articles for the *Medical Brief?* This journal is so largely patronized by the doctors of the cross roads and hamlet that it is one of the best advertising mediums in the world. Its proprietor has proven the demand for his wares by becoming rich beyond the dreams of avarice. Chiefly through his interest in the semi-proprietary preparations advertised in its columns, you will doubtless reply, but why should not a doctor editor profit by medical preparations? This much is certain, viz.—if the profession did not consume such preparations the editor of the *Brief* would have failed in business. And now for the character of the preparations upon which the fortune of the *Brief* man was built. Listerine, I believe, was the cornerstone. Who has not used it? Who has not carried grist to Dr. Lawrence's mill, simply because it is easier to read a label than to use our brains? Apropos, I was just reading some of the ads in the *Journal of the A. M. A.* "The Vibrator" that "gets the nerve endings and allows the operator to control nutrition to any part of the body." "Vapso-Cresolene, the atmistopharmacon for Whooping Cough"; "Iatrol"; "Topliff's suppositories for Hemorrhoids"; "Cactina Pellets," for the heart; "Seng," for the stomach; "Listerine"; "Lithiated Hydrangea"; "Ungentine"; "Tyree's Antiseptic Powder"; "Neoferrum"; "Stypticum," etc. Dr. "Ready-Made" seems to have a fair field to select from in our own *Journal of the A. M. A.* Let me see, I believe such things as "Midy Capsules" and "Scott's Emulsion" have appeared at various times in the *Journal.* Fig Syrup once adorned its pages. Many of these are to be found in the newspapers.

One would think that local pride should have prevented my friend, Dr. Wyeth, from betraying the fraud perpetrated upon the new York profession. Can it be possible that New York doctors could be cajoled into the belief that either the *New York Medical Journal* or the *Medical Record* would pay a stenographer $20 for a short article by anybody? I infer that the poor woman made better terms with the *Medical Brief.* If so, all honor to the journal that is willing to pay well for the stuff which a pampered medical press is wont to devour without so much as saying, "I thank you."

If the New York profession is so "easy" as Dr. Wyeth claims it is, New York should be a paradise for the vendor of medical gold bricks.

Would it be immodest for me to take the expression, "other reputable men," in your letter, Mr. Editor, as having a personal application? I have myself published short articles in the *Medical Brief*. My photograph, also, appeared in its columns once on a time. What about it, Mr. Editor? I am just about to send another short article to the same publication. Furthermore, oh, shameless confession! I wrote and sent the articles myself. I have no stenographic "Eve," mendicant otherwise, to lay the blame upon. I can not even be in fashion. Likewise did I send the photograph myself. Would that I could explain, as do some of my brethren when their pictures appear in the publications of the Philistines ,and say: "Somebody, at some time, stole my picture, which was published in some journal somewhere by somebody or other!" Alas! I have left my pictures around in all sorts of positions that should be tempting to the dishonest among literary folk, yet nobody has ever stolen one, and if they had, they wouldn't have published it, I'm sure. How I sympathize with those of the brethren who are more successful in having things stolen. How fatal the gift of beauty!

I have serious doubts as to my friend, Dr. Wyeth—really feeling ashamed of his alleged connection with the *Medical Brief*. He is broad gauged, liberal and a most charming personality. I suspect, however, that he yielded to the policy pressure of the multifarious medical snob. I do not see how he, a medical educator, could consistently administer a slap to the large number of subscribers to the *Medical Brief*. Surely he has never instructed his publishers not to sell his text-book to the "cross roads" doctor. Now, Mr. Editor, this is no defense of the *Medical* Brief and its ilk. The success of such journals proves the demand for them. When the profession is educated to a higher plane than that of the "what's good for measles" article, such journals will cease to exist—not before. I merely protest against "snobbishness," editorial or otherwise, and against the demand for explanation of, or apology for, literary conduct which is a matter of personal privilege. Variations of taste or policy are by no means criminal or unprofessional.

G. FRANK LYDSTON.

